# The impact of frailty on cognitive outcomes in elderly patients with post-stroke subjective cognitive complaints

**DOI:** 10.3389/fneur.2025.1701866

**Published:** 2025-11-19

**Authors:** Jiayu Duan, Yunling Zhang, Xianglan Jin, Shaozhen Ji

**Affiliations:** 1 Beijing University of Chinese Medicine, Beijing, China; 2 Department of Neurology, Dongfang Hospital, Beijing University of Chinese Medicine, Beijing, China; 3 Department of Neurology, China Academy of Chinese Medical Sciences Xiyuan Hospital, Beijing, China

**Keywords:** frailty, cognitive recovery, elder, stroke, post-stroke subjective cognitive complaints, association

## Abstract

**Background and purpose:**

Frailty delineates a state of poor health characterized by the accumulation of age-related health deficits, and was associated with cognitive decline in patients with subjective cognitive complaints (SCC). However, whether cognitive recovery is related to frailty in elderly patients with post-stroke SCC remained unknown. This study investigated cognitive outcomes in patients with SCC within 1 year after stroke, identified the relationship between frailty and cognitive recovery, and determined factors associated with cognitive recovery.

**Methods:**

Patients over the age of 60 with a clinical diagnosis of post-stroke SCC were included in this study, who had evidence of cognitive deficits, including Clinical Dementia Rating Scale (CDR) = 0.5, Montreal Cognitive Assessment (MoCA) score < 26, and Mini–Mental State Examination score > 17 (illiterate) or > 20 (primary school) or > 24 (junior school or above). A 32-item frailty index (FI) was operationalized using various data at baseline to measure frailty. Neuropsychological assessments were conducted at two time points: at baseline, which occurred within 2 weeks to 6 months of the stroke onset, and at the six-month follow-up. Cognitive recovery was operationalized as unimpaired cognition (MoCA score≥26 and CDR = 0) after 6 months. Factors associated with recovery were defined through logistic regression analysis.

**Results:**

After 6 months, 414 patients completed the follow-up with 53 (12.80%) presenting cognitive recovery. Contrary to expectations, frailty at baseline was not associated with cognitive recovery in patients with post-stroke SCC. A smaller proportion of women, higher baseline MoCA scores, and thalamus lesions were independently associated with high chance of cognitive recovery.

**Conclusion:**

This study found no association between baseline frailty and cognitive recovery at 6 months in post-stroke SCC patients. However, sex, higher baseline MoCA scores, and thalamic lesions independently predict cognitive function recovery in patients with SCC after stroke, which may influence the effectiveness of intervention measures.

## 1 Introduction

Subjective cognitive complaints (SCC) are prevalent following stroke, with prevalence rates ranging from 28.6 to 90.2%, contingent on SCC definitions, time since stroke, stroke characteristics, and the instruments employed (1). A significant number of studies examining post-stroke cognition have centered on objective assessment, while subjective cognitive complaints (SCC) have received comparatively less attention (2). Given that SCC was associated with objective neuropsychological test performance decline (3), it may be useful in identifying and diagnosing post-stroke cognitive impairment (PSCI). However, evidence has shown that the number of people with post-stroke SCC and the number of people with final PSCI are not entirely consistent (4). This discrepancy may be attributed to the time of screening or assessment of cognitive complaints, given that many patients with cognitive disorders after stroke will show recovery over time (1). Identifying potential factors that can predict cognitive recovery after stroke may facilitate a more profound comprehension of the etiology of PSCI, thereby offering guidance for diagnosis and the implementation of personalized rehabilitation interventions.

Frailty as deficit accumulation is a concept that delineates a state of poor health characterized by the accumulation of age-related health deficits (5). Some studies have found that frailty is associated with cognitive decline in patients with SCC (6, 7). In addition, a longitudinal study has found that frailty and SCC share a similar trajectory in older adults (8). However, due to the variety of scales used for the diagnosis of frailty, the heterogeneity of the studies is relatively high. Rockwood and colleagues describe the concept of frailty by measuring accumulated deficits across multiple systems, including comorbidity, physical function, nutritional status, and more (9). The calculation of a Frailty Index (FI) is predicated on the number of deficits observed, with higher indices denoting more pronounced frailty and a consequent prediction of adverse outcomes (10). FI is a multidomain tool based on comprehensive geriatric assessment that has been widely used in related research in recent years (11, 12). It is currently unclear whether cognitive recovery is related to FI scores in elderly patients with post-stroke SCC.

In the present longitudinal study, we investigated cognitive outcomes in patients with evidence of objective cognitive disorders who reported SCC in the early phase (within 6 months) after stroke and identified the relationship between frailty and cognitive recovery. The findings presented herein are derived from the “prospective multi-center cohort study of PSCI” data set (13).

## 2 Materials and methods

This study constituted a secondary examination of data pertaining to the “prospective multi-center cohort study of PSCI,” a study designed to establish an integrated health management network in China, predicated on home-based free-living intellectual and physical activities, with the objective of enhancing the health and welfare of stroke survivors. Research process and further details are described in the previous study (4). The present study was predicated on data procured at baseline and 6-month follow-up from the “prospective multicenter cohort study of PSCI.” The baseline survey was administered during 14 days to 6 months after acute stroke onset. The follow-up visit occurred after 6 months, employing the same evaluation method that entailed a structured clinical work-up and a standardized clinical history and assessments. Face-to-face interviews were conducted by trained physicians and nurses. Informed consent was obtained from all participants, and the study was approved by the research ethics committee of Dongfang Hospital prior to its initiation (approval number: 2011123004). The study was conducted in accordance with the World Medical Association Declaration of Helsinki.

### 2.1 Post-stroke SCC

The presence of post-stroke SCC was assessed using self-reports and the Checklist for Cognitive and Emotional Consequences Following Stroke (CLCE-24), a standardized interview exploring post-stroke cognitive, emotional, and behavioral complaints (14). The CLCE-24 comprises 13 cognitive, 9 emotional, and 2 non-specified items, which was scored on presence and interference in daily life: zero (SCC not present), one (doubtful presence), two (present, but not affecting daily life), three (present and negatively affecting daily life). Post-stroke SCC were defined as cognitive failures or problems worried and reported by patients themselves after the stroke incident, and at least one “present” or “doubtful presence” item as assessed by the CLCE-24.

### 2.2 Study population

Patients over the age of 60 with post-stroke SCC were included in this study, who had evidence of cognitive impairment at baseline. Specifically, this evidence manifested as a Clinical Dementia Rating (CDR) score = 0.5, a Montreal Cognitive Assessment (MoCA) score < 26, and a Mini-Mental State Examination (MMSE) score > 17 (illiterate) or > 20 (primary school) or > 24 (junior school and above). The exclusion criteria were as follows: indication of pre-stroke SCC; severe and major depression, or HAMD score ≥ 17; severe anxiety, aphasia, apraxia, agnosia, or other factors that might preclude completion of neuropsychological assessments; and other disorders or use of medication that might affect cognitive functions.

### 2.3 The frailty index

The FI is defined as a quantitative metric that calculates the presence or absence of each health deficit (10). In principle, the FI ranges from 0 to 1.0, as a proportion of the total (15). The FI exhibits a number of deficits that are weighted equally across different domains. It has been demonstrated that when a minimum of 30 variables is incorporated, FI functions as a reliable predictor of mortality (16). The FI in this study comprised 32 items (Supplementary Table 1), and was conducted post-hoc. Candidate variables were selected, screened, and scored in accordance with standard procedures (17). This study lacked information regarding the ability to walk 800 meters, as only information regarding walking ability over 200 meters was collected (11) (See Supplementary Table 1).

The FI score was computed using the coding demonstrated in the Supplementary Table 1. For patients with available coding on a minimum of 30 variables, the sum was divided by the number of codings. The resulting score ranged from 0.0 to 1.0, with higher scores indicating greater severity of frailty. Rockwood and colleagues have previously demonstrated in their research that 0.25 can be used as an empirical cutoff between robust and frail individuals (18). In this study, the participants were categorized as “no frail” if FI was below 0.24, and “frail” if FI was higher than 0.24, aligning with the previous research on frailty (18, 19).

### 2.4 Neuropsychological assessment

At the baseline, cognitive functioning and emotional status were documented. The MMSE, MoCA, and CDR were utilized to assess cognitive function, while the HAMD was employed to evaluate the status of depression subsequent to stroke. The capacity for daily living was gaged employing the Activities of Daily Living (ADL) scale. Cognitive recovery was delineated as a shift from cognitive impairment to unimpaired cognitive performance following a period of 6 months, as determined by MoCA score ≥ 26 and CDR = 0, with these evaluations conducted by at least one experienced neurologist.

### 2.5 Other data collection

Demographic characteristics, stroke characteristics, risk factors, and cognition were surveyed with structural questionnaires by trained physicians and nurses at baseline. The demographic characteristics encompassed age, sex, and years of education. The stroke characteristics that were examined included stroke severity, and the location and laterality of stroke lesions. The severity of the stroke was evaluated using the National Institutes of Health Stroke Scale (NIHSS). Stroke characteristics, including location and laterality, was derived from CT or MRI findings. Risk factors encompassed the consumption of tobacco and alcohol. A comprehensive array of factors was considered in the baseline survey, including but not limited to tea and coffee consumption and mobile phone usage patterns. The consumption of tobacco products, alcoholic beverages, and tea and coffee was defined as either current or former intake. The collection of information regarding mobile phone use was conducted on frequency (at least 30 min per day) based on a four-point scale (almost every day, 3–4 days per week, 1–2 days per week, and rarely).

### 2.6 Statistics

Continuous variables were presented as median (interquartile range (IQR)) and compared by Mann–Whitney U tests. Categorical variables were described as percentages and compared by chi-square tests and Fisher’s exact tests. Odds ratio (OR) was used to estimate the relative risk for cognitive recovery, with 95% confidence intervals (CI) employed to measure the precision of these estimates. Univariate and multivariable logistic regression analyses were conducted to ascertain the factors associated with cognitive recovery. The model 1 examines the relationship between frailty and cognitive recovery with univariate analyses. The model 2 consists of the strongest predictors of cognitive recovery, which were initially determined in characteristics analyses of patients with cognitive recovery vs. no cognitive recovery (*p* < 0.05). The model 3 was controlled for confounders associated with frailty or cognitive recovery, which were selected based on previous literature (20–23). The FI score can be either a categorical variable or a continuous variable (10). Therefore, the FI score was first treated as a categorical variable and then as a continuous variable in the logistic regression analyses.

All statistical tests were two-sided, and a *p* value < 0.05 was considered statistically significant. The collected data were then subjected to statistical analysis using SPSS version 26.0 (IBM Corp., Armonk, NY, USA).

## 3 Results

A total of 426 patients with post-stroke SCC have been included at baseline. Basic demographic and clinical characteristics are shown in Table 1. After 6 months, 12 individuals withdrew or were lost to follow-up (Supplementary Table 2). Finally, 414 (about 97.18%) samples were included in the final analyses (Supplementary Figure 1). Four hundred and fourteen participants (50.97% women, median/IQR age = 70.00/ (64.00, 76.00)) had both FI scores at baseline and completed neuropsychological assessment at 6 months in whom median/IQR FI score was 0.16/ (0.09, 0.22). Of these, 53 (12.80%) participants were assessed as cognitive recovery, and 361 (87.20%) as no cognitive recovery. The results after 6 months of follow-up showed that women had more difficulty achieving cognitive recovery than men (*p* < 0.01). The Cognitive recovery group had a smaller proportion of patients with cortical infarction (*p* = 0.04), a higher proportion of patients with thalamic infarction (*p* = 0.01), and higher MoCA scores at baseline (*p* < 0.01), but there were no differences in age and education level when compared to the No cognitive recovery group (Table 2).

**Table 1:** Baseline characteristics of patients with post-stroke SCC.

Characteristics	Entire sample *n* = 426	No Frail *n* = 342	Frail *n* = 84
Demographic characteristics			
Age, median (IQR), y	70.00 (64.00, 76.00)	70.00 (64.00, 76.00)	71.00 (65.00, 79.00)
Female sex, *n* (%)	215 (50.47)	172 (50.29)	43 (51.19)
Level of education, *n* (%)			
Low (years of education <12)	262 (61.50)	207 (60.53)	55 (65.48)
High (years of education ≥12)	164 (38.50)	135 (39.47)	29 (34.52)
Stroke characteristics			
NIHSS, median (IQR)	0.00 (0.00, 3.00)	0.00 (0.00, 2.00)	3.50 (1.00, 6.00) ^*^
Location, *n* (%)			
Cerebral cortex	40 (9.39)	27 (7.89)	13 (15.48) ^*^
Subcortex	108 (25.35)	79 (23.10)	29 (34.52) ^*^
Basal ganglia	287 (67.37)	238 (69.59)	49 (58.33) ^*^
Thalamus	31 (7.28)	23 (6.73)	8 (9.52)
Brain stem	33 (7.75)	24 (7.02)	9 (10.71)
Cerebellum	15 (3.52)	12 (3.51)	3 (3.57)
Laterality, *n* (%)			
Left hemisphere	91 (21.36)	76 (22.22)	15 (17.86)
Right hemisphere	82 (19.25)	61 (17.84)	21 (25.00)
Bilateral hemispheres	231 (54.23)	184 (53.80)	47 (55.95)
Risk factors			
Smoking, *n* (%)	122 (28.64)	93 (27.19)	29 (34.52)
Alcohol intake, *n* (%)	108 (25.35)	89 (26.02)	19 (22.62)
Cognition			
MoCA score, median (IQR)	23.00 (20.00, 24.00)	23.00 (21.00, 24.00)	21.00 (18.00, 23.00) ^*^
Other			
Tea intake, *n* (%)	269 (63.15)	218 (63.74)	51 (60.71)
Coffee intake, *n* (%)	11 (2.58)	9 (2.63)	2 (2.38)
Mobile-phone use, *n* (%)	288 (67.61)	237 (69.30)	51 (60.71)

SCC, Subjective Cognitive Complaints; NIHSS, National Institute of Health Stroke Scale; MoCA, Montreal Cognitive Assessment; IQR, Interquartile range; ^*^, *p* < 0.05.

**Table 2:** Characteristics of patients with cognitive recovery vs. no cognitive recovery.

Characteristics	Cognitive recovery *n* = 53	No cognitive recovery *n* = 361	*p*-value
Demographic characteristics			
Age, median (IQR), y	68.00 (63.50, 76.00)	70.00 (64.00, 77.00)	0.52
Female sex, *n* (%)	13 (24.53)	198 (54.85)	< 0.01
Level of education, *n* (%)			0.06
Low (years of education <12)	26 (49.06)	226 (62.60)	
High (years of education ≥12)	27 (50.94)	135 (37.40)	
Stroke characteristics			
NIHSS, median (IQR)	1.00 (0.00, 3.00)	0.00 (0.00, 3.00)	0.49
Location, *n* (%)			
Cerebral cortex	1 (1.89)	39 (10.80)	0.04
Subcortex	14 (26.42)	91 (25.21)	0.85
Basal ganglia	37 (69.81)	242 (67.04)	0.69
Thalamus	9 (16.98)	22 (6.09)	0.01
Brain stem	1 (1.89)	28 (7.76)	0.15
Cerebellum	2 (3.77)	12 (3.32)	0.70
Laterality, *n* (%)			0.15
Left hemisphere	17 (32.08)	72 (19.94)	
Right hemisphere	8 (15.09)	74 (20.50)	
Bilateral hemispheres	24 (45.28)	197 (54.57)	
Risk factors			
Smoking, *n* (%)	18 (33.96)	99 (27.42)	0.32
Alcohol intake, *n* (%)	15 (28.30)	89 (24.65)	0.57
Cognition			
MoCA score at baseline, median (IQR)	24.00 (23.00, 25.00)	22.00 (20.00, 24.00)	< 0.01
Other			
Tea intake, *n* (%)	32 (60.38)	230 (63.71)	0.64
Coffee intake, *n* (%)	3 (5.66)	8 (2.22)	0.16
Mobile-phone use, *n* (%)	38 (71.70)	242 (67.04)	0.50
Frailty, *n* (%)	7 (13.21)	73 (20.22)	0.23

NIHSS, National Institute of Health Stroke Scale; MoCA, Montreal Cognitive Assessment; IQR, Interquartile range.

In univariate analyses, contrary to expectations, whether patients with post-stroke SCC were frail at baseline was not associated with cognitive recovery. In multivariate analysis, four possible factors associated with cognitive recovery in patients with post-stroke SCC were sex, the MoCA score at baseline, and lesions locations (cerebral cortex and thalamus). These four factors and whether post-stroke SCC patients were frail at baseline were selected, ultimately forming the model 2 containing five variables. The results showed that sex, MoCA score at baseline, and thalamic lesions were still associated with cognitive recovery, while whether post-stroke SCC patients were frail at baseline was not. The associations remained statistically significant after controlling for a variety of confounders in the model 3 (Table 3). The study subsequently performed a regression analysis with FI as a continuous variable. The results were similar to those obtained previously: sex, MoCA score at baseline, and thalamic lesions were associated with cognitive recovery, while frailty degree was still not (Table 4).

**Table 3:** Frail and No Frail and other factors associated with cognitive recovery in patients with post-stroke SCC.

Factors	OR (95% CI)	*P*-value
Model 1^a^		
No Frail	Reference group	
Frail	0.600 (0.260–1.385)	0.23
Model 2^b^		
No Frail	Reference group	
Frail	0.916 (0.368–2.280)	0.85
Female sex	0.308 (0.156–0.610)	< 0.01
Location		
Cerebral cortex	0.162 (0.021–1.250)	0.08
Thalamus	2.727 (1.068–6.962)	0.04
MoCA score at baseline	1.340 (1.137–1.580)	< 0.01
Model 3^c^		
No Frail	Reference group	
Frail	0.651 (0.231–1.834)	0.42
Female sex	0.310 (0.156–0.616)	< 0.01
NIHSS	1.110 (0.953–1.293)	0.18
Location		
Cerebral cortex	0.164 (0.021–1.300)	0.09
Subcortex	1.461 (0.704–3.033)	0.31
Basal ganglia	1.136 (0.563–2.291)	0.72
Thalamus	3.091 (1.182–8.082)	0.02
MoCA score at baseline	1.360 (1.152–1.605)	< 0.01

a Model 1: univariate model.

b Model 2: adjusted for sex, MoCA score at baseline, lesions location (cerebral cortex and thalamus).

c Model 3: adjusted for the variables in Model 2 plus all other comorbidities listed in Tables 1, 2.

SCC, Subjective cognitive complaints; MoCA, Montreal Cognitive Assessment; NIHSS, National Institute of Health Stroke Scale; CI, confidence interval; OR, odds ratio.

**Table 4:** Frailty degree and other factors associated with cognitive recovery in patients with post-stroke SCC.

Factors	OR (95% CI)	*p*-value
Model 1^a^		
Frail degree	0.468 (0.048–4.536)	0.51
Model 2^b^		
Frail degree	2.476 (0.208–29.435)	0.47
Female sex	0.304 (0.153–0.602)	< 0.01
Location		
Cerebral cortex	0.151 (0.020–1.167)	0.07
Thalamus	2.707 (1.069–6.855)	0.04
MoCA score at baseline	1.364 (1.153–1.614)	< 0.01
Model 3^c^		
Frail degree	1.270 (0.067–24.031)	0.87
Female sex	0.305 (0.153–0.607)	< 0.01
NIHSS	1.072 (0.917–1.253)	0.39
Location		
Cerebral cortex	0.156 (0.020–1.240)	0.08
Subcortex	1.439 (0.694–2.983)	0.33
Basal ganglia	1.137 (0.563–2.294)	0.72
Thalamus	0.341 (0.132–0.883)	0.03
MoCA score at baseline	1.376 (1.162–1.631)	< 0.01

a Model 1: univariate model.

b Model 2: adjusted for sex, MoCA score at baseline, lesions location (cerebral cortex and thalamus).

c Model 3: adjusted for the variables in Model 2 plus all other comorbidities listed in Tables 1, 2.

SCC, Subjective cognitive complaints; MoCA, Montreal Cognitive Assessment; NIHSS, National Institute of Health Stroke Scale; CI, confidence interval; OR, odds ratio.

## 4 Discussion

There have been some studies investigating cognitive trajectories following stroke (24, 25), in which few focused on cognitive function recovery in survivors with stroke. This study showed that improvement of objective cognitive function did indeed take place in some older patients with post-stroke SCC after 1 year. Although some pervious researches demonstrated that frailty might be associated with cognition deterioration in elders after stroke (26, 27), but this study did not find a significant influence on cognition recovery in patients aged over 60 years old who had post-stroke SCC. In addition, sex, baseline MoCA scores, and thalamic lesions might influence the recovery of cognitive function in survivors with SCC after stroke.

Frailty targets the elderly population and is related to aging (28). It’s reported that frailty is closely correlated to cognitive decline in patients with aged-related diseases such as Alzheimer’s disease (AD) (29) and Parkinson Disease (PD) (30). Recent studies have identified that frailty was related to both odds of AD and disease expression (29), which might result from its influence in AD biomarkers (31), decreasing patients’ cognitive resilience and accelerating cognitive dysfunction due to abnormal AD-related pathology (32). The change trajectory of cognitive function after stroke is different from that in AD, and the initial post stroke period (up to 1 year) appears to be accompanied by cognitive recovery (33). The characteristics of the trajectory of cognition after stroke and the timeliness of cognitive recovery were taken into account in this study, we observed cognitive recovery within 1 year after stroke event. No significate correlation was observed between frailty and cognitive recovery in old patients with post-stroke SCC, which might be contributed by the pathology of PSCI including vascular dysregulation (34), inflammation (35), and functional connectivity (36), differing from AD’s.

Post-stroke SCC is reported to be associated with psychological factors such as depression, anxiety, perceived stress and coping style (37). The results of this study demonstrated that objective cognitive performance exhibited improvement following a stroke in 12.8% of elders with post-stroke SCC, which suggested that psychological distress due to stroke incident might affect acute deficiencies in cognitive test scores or immediate cognitive syndromes in patients with post-stroke SCC. Negative results in correlation between frailty and cognitive recovery might be due to that SCC under the influence of stroke incident may not be closely associated with objective frailty. This study defined cognitive recovery as a MoCA score ≥ 26 and CDR = 0 at the 6-month follow-up. This criterion is relatively stringent, as some post-stroke patients may show improvement but remain below this threshold, which may account for the negative results. Additionally, the average age of elderly patients included in this study was relatively young (70.71 ± 7.35 years), with a predominance of mild strokes, resulting in relatively low frailty levels (median FI = 0.16). This may support another explanation for uncorrelation between frailty and cognitive recovery. Moreover, this study employed FI to evaluate the frailty in patients. FI, a multifaceted evaluation instrument that appraises the severity of frailty in geriatric patients by assessing numerous systems, including comorbidity, physical function, and nutritional status, is a widely utilized metric (9). The definition of frailty in FI is more objective and focuses on physiological load (38) rather than psychological frailty (39). FI struggles to capture the psychological/social vulnerability domains associated with SCC. Furthermore, social vulnerabilities, such as an absence of sufficient social connections, support, or interaction, are also salient factors contributing to physical decline and cognitive impairment in older adults (40), which are difficult to capture in FI. Therefore, when conducting frailty assessments of older adults in the future, it is important to consider adding assessments of social and psychological factors to the objective FI to better reflect the overall state of aging in elderly patients.

The results of this study showed that a lower proportion of female subjects, higher MoCA scores at baseline, and thalamic lesions were independently associated with cognitive recovery at 6 months after stroke. The proportion of women who achieve cognitive recovery after stroke is lower, which may not be due to gender alone, but rather because women in the general population who experience stroke tend to be older, have fewer years of education (i.e., lower cognitive reserve), and have more severe strokes or poorer cognitive function prior to stroke (41, 42). These disadvantages faced by women result in fewer individuals ultimately regaining their cognitive abilities. Low education level caused that women primarily assumed household responsibilities and lacked opportunities for vocational training and complex cognitive activities. These factors collectively contributed to women experiencing greater difficulty in achieving cognitive recovery. Furthermore, women’s SCC are more predictive of future objective cognitive change (43) and are more strongly associated with subsequent dementia (44), indicating that women’s self-assessment may more closely reflect their actual cognitive changes. In addition, despite adjusting for multiple covariates, residual confounding due to measurement errors or unmeasured factors. This result suggested delve deeper was needed into the impact of gender on cognitive recovery following stroke. This study revealed that patients who reported SCC following stroke yet exhibited higher MoCA scores at baseline demonstrated a greater probability of cognitive recovery. While some studies have indicated an association between the evlution of post-stroke SCC and psychological resilience (37), the results of this study suggest a role for baseline cognitive performance in the improvement of objective cognitive function, which is consistent with previous studies (4). This may be related to factors such as milder disease severity, greater brain network plasticity, and richer cognitive reserve in patients with higher baseline MoCA scores (45–47). Numerous studies have consistently shown that although thalamic stroke patients show improvement in the early stages after stroke (48), they may still experience acute and long-term cognitive impairment (49, 50), which may be related to damage to frontal lobe-related functions. This study found that thalamic infarction is associated with cognitive function recovery in the early stage of stroke in patients with SCC after stroke. This may be due to the high neuroplasticity of the thalamus (51) and the fact that the infarct area was not located in the anterior region in almost all patients (49, 50). However, due to the lack of long-term research and detailed neuroimaging data including size, vascular distribution areas, imaging modalities, it is currently impossible to provide a reasonable explanation for this observation. Future studies should use more clearly report lesion classification methods to further explore the impact of thalamic lesions on cognitive recovery in stroke patients.

Although the current study is one of the few to examine the relationship between frailty and cognitive recovery, making it more aligned with clinical needs, it has several limitations. First, although the number of subjects in the PSCI study was quite large, the sample size was still insufficient for exploring factors related to cognitive function recovery in patients with SCC after stroke. Second, the findings were derived from data collected from participants who did not exhibit severe symptoms of depression and anxiety. This was due to the interaction between post-stroke SCC and mood (depression and anxiety) (52, 53). However, the association between the severity of post-stroke SCC, two components of SCC (SCC-content vs. SCC-worry), and cognitive recovery was not investigated. Third, the sample demonstrated overall low levels of frailty, with a median FI at baseline of 0.16, which may explain why no association was found between frailty and cognitive recovery at follow-up. Fourth, the generalizability of the study findings may be more applicable to patients who have experienced mild stroke, as the baseline characteristics of the study subjects were comparable. Fifth, this study employed simple cognitive screening measures to assess and define cognitive recovery. Future research utilizing more thorough neuropsychological assessments at baseline and follow-up stages may yield different results (54, 55). Moreover, the odds ratios for FI are unstable with wide confidence intervals, which may lead to potential model instability and limited statistical power. Although multiple covariates had been adjusted, residual confounding due to measurement errors or unmeasured factors (such as treatment factors, lesion size, and recurrent stroke) could not be avoided.

## 5 Conclusion

A subset of patients diagnosed with SCC at an early stage following a stroke demonstrated cognitive recovery. Although previous studies have suggested that the frailty in older adults is associated with cognitive decline after stroke, the present study found no association between baseline frailty and cognitive recovery at 6 months in post-stroke SCC patients. However, this study found that certain factors appear to independently predict cognitive function recovery in patients with SCC after stroke, including sex, higher baseline MoCA scores, and thalamic lesions, which may influence the effectiveness of intervention measures.

## Data Availability

The raw data supporting the conclusions of this article will be made available by the authors, without undue reservation.
