# Supplementary material for: The impact of frailty on cognitive outcomes in elderly patients with post-stroke subjective cognitive complaints
Source: Front Neurol. 2025 Nov 19;16:1701866. doi: 10.3389/fneur.2025.1701866 (PMC12672352; doi:10.3389/fneur.2025.1701866)
Supplement: Supplementary file 1 [file Data_Sheet_1.pdf]

Supplementary Figure 1 Flow-chart of participants flow

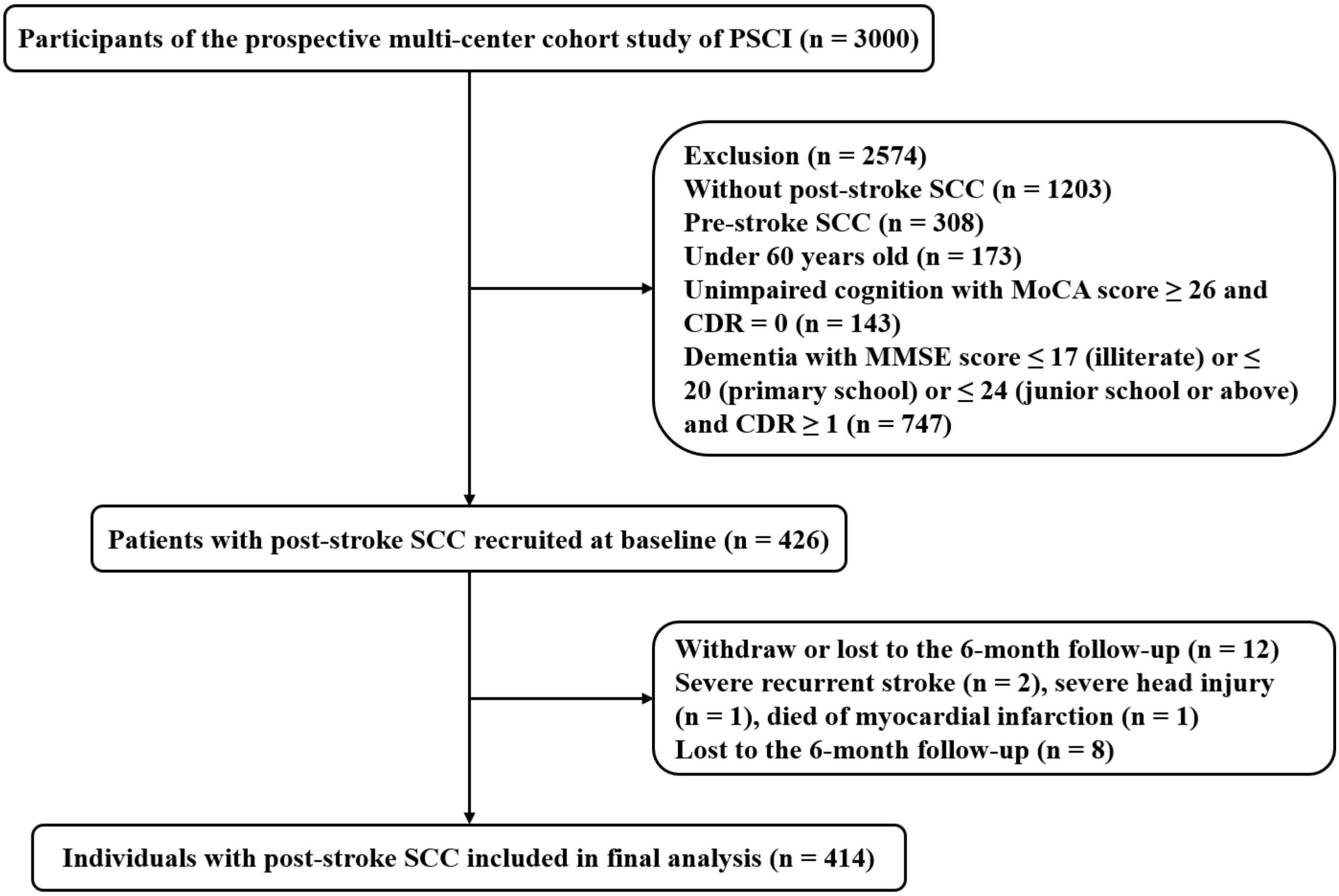

Abbreviations: PSCI: Post-stroke cognitive impairment; SCC: Subjective Cognitive Complaints; MoCA: Montreal Cognitive Assessment; MMSE: Mini–Mental State Examination; CDR: Clinical Dementia Rating Scale.
