# Supplementary material for: The impact of frailty on cognitive outcomes in elderly patients with post-stroke subjective cognitive complaints
Source: Front Neurol. 2025 Nov 19;16:1701866. doi: 10.3389/fneur.2025.1701866 (PMC12672352; doi:10.3389/fneur.2025.1701866)
Supplement: Supplementary file 2 [file Table_1.docx]

Supplementary Table 1 Comprehensive Geriatric Assessment-Based Frailty Index

| Domain | Deficit coding | n (%) |
| --- | --- | --- |
| *Medical history* |  |  |
| Stroke characteristics |  |  |
| Lacunar infarction | no = 0, yes = 1 | 251 (60.63) |
| Hemorrhagic stroke | no = 0, yes = 1 | 13 (3.14) |
| Comorbidities and risk factors |  |  |
| Hypertension | no = 0, yes = 1 | 295 (71.26) |
| Diabetes | no = 0, yes = 1 | 105 (25.36) |
| Heart disease | no = 0, yes = 1 | 83 (20.05) |
| Hyperlipidemias | no = 0, yes = 1 | 93 (22.46) |
| Previous TIA/stroke | no = 0, yes = 1 | 80 (19.32) |
| Medications |  |  |
| Polypharmacy | no = 0, yes = 1 | 266 (64.25) |
| *Functional limitations* |  |  |
| ADL items |  |  |
| Need personal help for using transportation | no = 0, yes = 1 | 45 (10.87) |
| Need personal help for preparing own meals | no = 0, yes = 1 | 53 (12.80) |
| Need personal help for housework | no = 0, yes = 1 | 36 (8.70) |
| Need personal help for taking own medications | no = 0, yes = 1 | 17 (4.11) |
| Need personal help for feeding | no = 0, yes = 1 | 14 (3.38) |
| Need personal help for dressing and undressing | no = 0, yes = 1 | 21 (5.07) |
| Need personal help for grooming | no = 0, yes = 1 | 21 (5.07) |
| Need personal help for washing your own clothes | no = 0, yes = 1 | 45 (10.87) |
| Need personal help for indoor activities | no = 0, yes = 1 | 16 (3.86) |
| Need personal help for getting in and out of bed | no = 0, yes = 1 | 17 (4.11) |
| Need personal help for bathing or shower | no = 0, yes = 1 | 57 (13.77) |
| Need personal help for using toilet | no = 0, yes = 1 | 39 (9.42) |
| Need personal help for cutting toenails | no = 0, yes = 1 | 40 (9.66) |
| Need personal help for shopping | no = 0, yes = 1 | 56 (13.53) |
| Need personal help for bowel and bladder control | no = 0, yes = 1 | 19 (4.59) |
| Need personal help for using telephone | no = 0, yes = 1 | 24 (5.80) |
| Need personal help for managing own money or paying bills | no = 0, yes = 1 | 46 (11.11) |
| Need personal help for staying home alone | no = 0, yes = 1 | 29 (7.00) |
| *Rosow-Bresau items* |  |  |
| Unable to walk up or down stairs | no = 0, yes = 1 | 37 (8.94) |
| Unable to walk 200 m (changed from unable to walk 800 m) | no = 0, yes = 1 | 26 (6.28) |
| *Other measures* |  |  |
| HAMD score | ≤ 7 = 0, > 7 = 1 | 68 (16.43) |
| Physical exercise | ≥ 1 h per day = 0, < 1 h per day = 1 | 330 (79.71) |
| BMI | 18.5-24.9 = 0, < 18.5 or ≥ 25.0 = 1 | 194 (46.86) |
| weight loss (Unintentional weight loss >4.5 kg in the past six month) | no = 0, yes = 1 | 7 (1.69) |

Abbreviations: TIA: transient ischemic attack; ADL: Activities of Daily Living; HAMD: Hamilton Depression Rating Scale; BMI: Body Mass Index.
