# Supplementary material for: The impact of frailty on cognitive outcomes in elderly patients with post-stroke subjective cognitive complaints
Source: Front Neurol. 2025 Nov 19;16:1701866. doi: 10.3389/fneur.2025.1701866 (PMC12672352; doi:10.3389/fneur.2025.1701866)
Supplement: Supplementary file 3 [file Table_2.docx]

Supplementary Table 2 Characteristics of subjects included vs not included in final analyses

|  | Final sample (n = 414) | Lost to follow-up (n = 12) | *P* value |
| --- | --- | --- | --- |
| Demographic characteristics |  |  |  |
| Age, median (IQR), y | 70.00 (64.00, 76.00) | 72.50 (67.00, 78.50) | 0.276 |
| Female sex, n (%) | 211 (50.97) | 4 (33.33) | 0.228 |
| Level of education, n (%) |  |  | 0.141 |
| Low (years of education <12) | 252 (60.87) | 10 (83.33) |  |
| High (years of education ≥12) | 162 (39.13) | 2 (16.67) |  |
| Stroke characteristics |  |  |  |
| NIHSS, median (IQR) | 0.00 (0.00, 3.00) | 0.50 (0.00, 2.50) | 0.980 |
| Location, n (%) |  |  |  |
| Cerebral cortex | 40 (9.66) | 0 (0.00) | 0.614 |
| Subcortex | 105 (25.36) | 3 (25.00) | 1.000 |
| Basal ganglia | 279 (67.39) | 8 (66.67) | 1.000 |
| Thalamus | 31 (7.49) | 0 (0.00) | 1.000 |
| Brain stem | 29 (7.00) | 4 (33.33) | 0.010 |
| Cerebellum | 14 (3.38) | 1 (8.33) | 0.353 |
| Laterality, n (%) |  |  |  |
| Left hemisphere | 89 (21.50) | 2 (16.67) | 1.000 |
| Right hemisphere | 82 (19.81) | 0 (0.00) | 0.134 |
| Bilateral hemispheres | 221 (53.38) | 10 (83.33) | 0.040 |
| Risk factors |  |  |  |
| Smoking, n (%) | 117 (28.26) | 5 (41.67) | 0.337 |
| Alcohol intake, n (%) | 104 (25.12) | 4 (33.33) | 0.509 |
| Cognition | | | |
| MoCA score, median (IQR) | 23.00 (20.00, 24.00) | 22.50 (18.50, 24.00) | 0.437 |
| Other |  |  |  |
| Tea intake, n (%) | 262 (63.29) | 7 (58.33) | 0.766 |
| Coffee intake, n (%) | 11 (2.66) | 0 (0.00) | 1.000 |
| Mobile-phone use, n (%) | 280 (67.63) | 8 (66.67) | 1.000 |

Abbreviations: NIHSS: National Institute of Health Stroke Scale; MoCA: Montreal Cognitive Assessment; IQR: Interquartile range.
